# Cultivar-dependent regulation of cytokinin biosynthesis in wheat: developmental expression of *TaIPT* genes and hormonal crosstalk during reproductive development

**DOI:** 10.1186/s12870-026-09374-0

**Published:** 2026-06-29

**Authors:** Joanna Bocian, Karolina Szala, Bartosz Jabłoński, Adnan Iqbal, Andrzej Bajguz, Magdalena Chmur, Anna Nadolska-Orczyk

**Affiliations:** 1 Plant Breeding and Acclimatization Institute – National Research Institute, Radzikow, Blonie, 05-870 Poland; 2 Department of Biology and Plant Ecology, Faculty of Biology, University of Bialystok, Ciolkowskiego 1J, Bialystok, 15-245 Poland

**Keywords:** *Triticum aestivum*, Cytokinin biosynthesis, *TaIPT* gene family, Phytohormones, Cytokinin–ABA interaction, Reproductive development, Wheat spike

## Abstract

**Background:**

Cytokinins are key regulators of plant growth, reproductive development, and yield formation. In cereals, cytokinin biosynthesis is catalyzed by isopentenyltransferase (IPT) enzymes, yet the genomic organization and developmental regulation of *IPT* genes in polyploid wheat remain incompletely understood, especially at the cultivar level.

**Results:**

Here, we present an integrated genomic, transcriptional, and hormonal analysis of the *TaIPT* gene family during vegetative and reproductive development in two wheat cultivars, awnless Kontesa and awned Ostka. Genome-wide analysis identified nine core *TaIPT* genes represented by 25 homoeologs distributed across the A, B, and D subgenomes, for which a unified nomenclature was established. Phylogenetic analysis resolved TaIPTs into conserved evolutionary clades corresponding to ATP/ADP-dependent and tRNA-dependent IPT groups.

Expression profiling revealed distinct spatial and temporal patterns of *TaIPT* transcription across roots, leaves, inflorescences, and developing spikes. Several *TaIPT* genes showed enhanced expression during early reproductive stages, coinciding with dynamic changes in cytokinin concentrations. Comparative analyses revealed cultivar-specific expression and co-variation patterns, with Kontesa displaying more compartmentalized *TaIPT* expression and Ostka showing coordinated activation of multiple *TaIPT* genes during early grain development. Hormone profiling further indicated stage-dependent associations between *TaIPT* expression, cytokinin metabolism, and the balance between cytokinins and abscisic acid. These relationships are interpreted as correlative and provide a framework for future functional testing rather than direct evidence of causality.

**Conclusions:**

Together, these results provide a cultivar-focused framework for understanding the organization and regulation of cytokinin biosynthesis genes in wheat. The data highlight cultivar-dependent *TaIPT* expression patterns and their association with cytokinin dynamics during reproductive development, while also identifying the need for homoeolog-specific and functional validation. This study establishes a foundation for future research on cytokinin-mediated regulation of wheat growth and grain development.

**Supplementary Information:**

The online version contains supplementary material available at https://doi.org/10.1186/s12870-026-09374-0.

## Highlights

Integrated genomic, expression, and hormone analyses reveal cultivar-specific regulation of cytokinin biosynthesis by a unified set of *TaIPT* genes and their coordinated crosstalk with ABA during wheat reproductive development.

**Supplementary Information:**

The online version contains supplementary material available at https://doi.org/10.1186/s12870-026-09374-0.

## Introduction

Common wheat (*Triticum aestivum* L., AABBDD) is one of the world’s most important cereal food and feed crops, and its continuous genetic improvement remains essential for global food security. Recent advances in New Genomic Techniques (NGTs), particularly CRISPR/Cas-based genome editing, have created unprecedented opportunities to optimize complex polyploid genomes for desirable agronomic traits [1–3]. A prerequisite for such targeted approaches is the precise identification and functional characterization of candidate genes and regulatory pathways controlling plant development and yield formation.

Phytohormones are central regulators of plant growth and development, with cytokinins (CKs) playing pivotal roles in root and shoot architecture, leaf senescence, stress responses, and grain yield in cereals [4–11] and other crops [12–16]. Cytokinin function is tightly integrated with other hormonal pathways, including auxins and abscisic acid (ABA), enabling coordinated regulation of developmental and environmental responses [17–20]. In wheat, elevated levels of *cis*-zeatin (cZ) have been shown to correlate positively with ABA accumulation, highlighting hormonally integrated control during reproductive development [7].

Among cytokinins, isoprenoid forms, *N*⁶-(Δ²-isopentenyl)adenine (iP), *trans*-zeatin (tZ), *cis*-zeatin (cZ), and dihydrozeatin (DZ) represent the most abundant and biologically active CKs, with tZ given the primary role in development [21–24]. For instance, tZ-type cytokinins regulate both root and shoot growth in monocotyledonous rice [5]. Field studies in wheat have identified more than twenty-five isoprenoid cytokinin metabolites, with free-base tZ and cZ predominating during early kernel development and positively correlating with yield potential [6, 7, 25]. These observations underscore the importance of precise regulation of cytokinin biosynthesis during key developmental transitions.

Cytokinin homeostasis is controlled by biosynthesis, metabolic conversion, irreversible degradation, and signal perception [26]. In the ATP/ADP-dependent biosynthetic pathway, adenosine phosphate–isopentenyltransferases (IPTs) catalyze the initial and rate-limiting step of cytokinin biosynthesis by prenylating ATP or ADP precursors, which are subsequently converted to active free bases by LONELY GUY (LOG) enzymes or hydroxylated by CYP735A monooxygenases [27–31]. Functional relevance of this pathway has been demonstrated in cereals, where altered LOG-Like Protein 6 activity affects awn length and grain yield in rice [32].

IPT enzymes are encoded by members of the *IPT* gene family, which is subdivided into ATP/ADP-IPTs responsible for iP- and tZ-type cytokinin biosynthesis, and tRNA-IPTs associated with cZ production [17, 33–35]. While the *IPT* gene family has been well characterized in model species and diploid crops, including cereals [17, 36, 37], its organization and regulation in polyploid wheat remain poorly understood. Wheat contains at least twenty-four *IPT* genes [38], many of which are present as homoeologous copies across the A, B, and D subgenomes. In polyploid cereals, homoeologs may display conserved or divergent expression patterns [39–42], influenced by *cis*-regulatory variation and transposable element insertions, with potential consequences for developmental regulation and yield-related traits [41–43].

Spatial and temporal regulation of *IPT* gene expression is a critical determinant of cytokinin-mediated developmental outcomes. In several plant species, *IPT* family members exhibit pronounced tissue- and stage-specific expression patterns [17, 33, 36, 44]. In wheat, coordinated regulation of selected *TaIPT* genes has been reported during reproductive development in spikes, grains, and flag leaves [44] indicating complex developmental control of cytokinin biosynthesis. Additionally, *IPT* genes have been implicated in modulating plant stress responses and yield in various species [33, 45]. In wheat, drought-induced expression of *IPT* genes has been associated with reduced grain yield [38, 46]. Moreover, altered expression of *TaIPT8* affects cytokinin levels and drought tolerance in wheat [38].

Recent hormonal studies demonstrate that CK, ABA, and auxin exhibit highly coordinated but contrasting temporal dynamics during grain filling, each contributing uniquely to sink establishment, starch accumulation, and senescence timing, which is reviewed in Zarea, 2025 [19]. However, the upstream regulatory architecture controlling these hormone trajectories in wheat remains poorly defined, especially in the context of polyploid-specific *IPT* gene families.

Our previous work has focused on cytokinin degradation pathways mediated by *TaCKX* genes and their regulation by NAC transcription factors [47–53] as well as establishing reference genes for RT-qPCR normalization for expression studies [54] in two wheat cultivars, awnless Kontesa and awned Ostka. Recent reviews and integrative studies emphasize that cytokinin-dependent regulation of cereal reproductive development involves not only biosynthesis, but also activation, degradation, conjugation, transport, and interaction with ABA, nitrogen status and cell-type-specific responses [19, 24, 26, 33, 55, 56]. In contrast, a cultivar-level analysis linking the unified *TaIPT* gene framework with developmental expression and endogenous cytokinin/ABA profiles is still lacking. The central research question of this study was whether the polyploid *TaIPT* gene family is regulated as a uniform cytokinin-biosynthetic module during wheat reproductive development or whether its expression, association with cytokinin pools, and interaction with ABA are organized in a cultivar-specific manner. We hypothesized that Kontesa and Ostka differ not only in the abundance of individual *TaIPT* transcripts, but also in the coordination between *TaIPT* expression, cytokinin metabolism, and cytokinin-ABA balance during the transition from anthesis to early grain development. To test this hypothesis, we combined phylogenetic analysis, detailed spatiotemporal expression profiling, and quantification of cytokinins and ABA in the two cultivars. This integrated approach was designed to move beyond gene-family annotation and to define cultivar-specific transcriptional-hormonal associations underlying wheat reproductive development.

## Materials and methods

### Plant material and growth conditions

Two spring wheat (*Triticum aestivum* L.) cultivars, Kontesa and Ostka, were used. These cultivars were selected because they differ in spike morphology and have been used previously in studies of cytokinin metabolism and developmental gene expression, enabling comparison with earlier *TaCKX*, *TaNAC* and reference-gene datasets. Kontesa is an older, traditional awnless spring wheat cultivar classified as a bread-quality cultivar (quality group B), characterized by relatively early heading and maturity and broad adaptation to Polish growing conditions, although its yield potential is lower than that of more recent high-yielding cultivars. Ostka (Ostka Smolicka) is an awned spring/facultative wheat cultivar classified as a quality cultivar (quality group A), with high grain-quality parameters, medium plant height, good lodging and pre-harvest sprouting resistance, tolerance to spring frosts, and suitability for intensive cultivation and late autumn sowing under favorable conditions. The presence of awns in Ostka is also agronomically relevant in areas exposed to wildlife pressure. Thus, the two cultivars differ not only in awn morphology but also in broader agronomic background, which was considered when interpreting cultivar-dependent hormonal and transcriptional differences.

Seeds were checked for uniformity and pre-germinated for 2 days at 4 °C and 5 days at room temperature in darkness, and transferred to soil under long-day conditions (16 h light/20°C; 8 h dark/18°C) as described previously [54]. Plants were grown under the same controlled conditions and sampled at comparable developmental stages. Tissues were collected in three independent biological replicates, each consisting of material pooled from separate plants, from 5 day-old seedling roots, fully expanded leaves (4-week plants), immature inflorescences (5–6 cm long), flag leaves subtending inflorescence (FL inflor.), developing spikes at 0, 4, 7, and 14 days after pollination (DAP), and the corresponding flag leaves (FL 0 DAP, FL 4 DAP, FL 7 DAP, FL 14 DAP). To ensure developmental uniformity of the sampled spike tissues, only the middle section of each spike was collected for analysis. The four spike time points were selected to cover the transition from anthesis through early grain development; they provide a developmental overview but do not resolve all short-term hormonal fluctuations. Samples were frozen in liquid nitrogen and stored at − 80 °C.

### Identification and in silico characterization of TaIPT genes

**Fig. 1:**
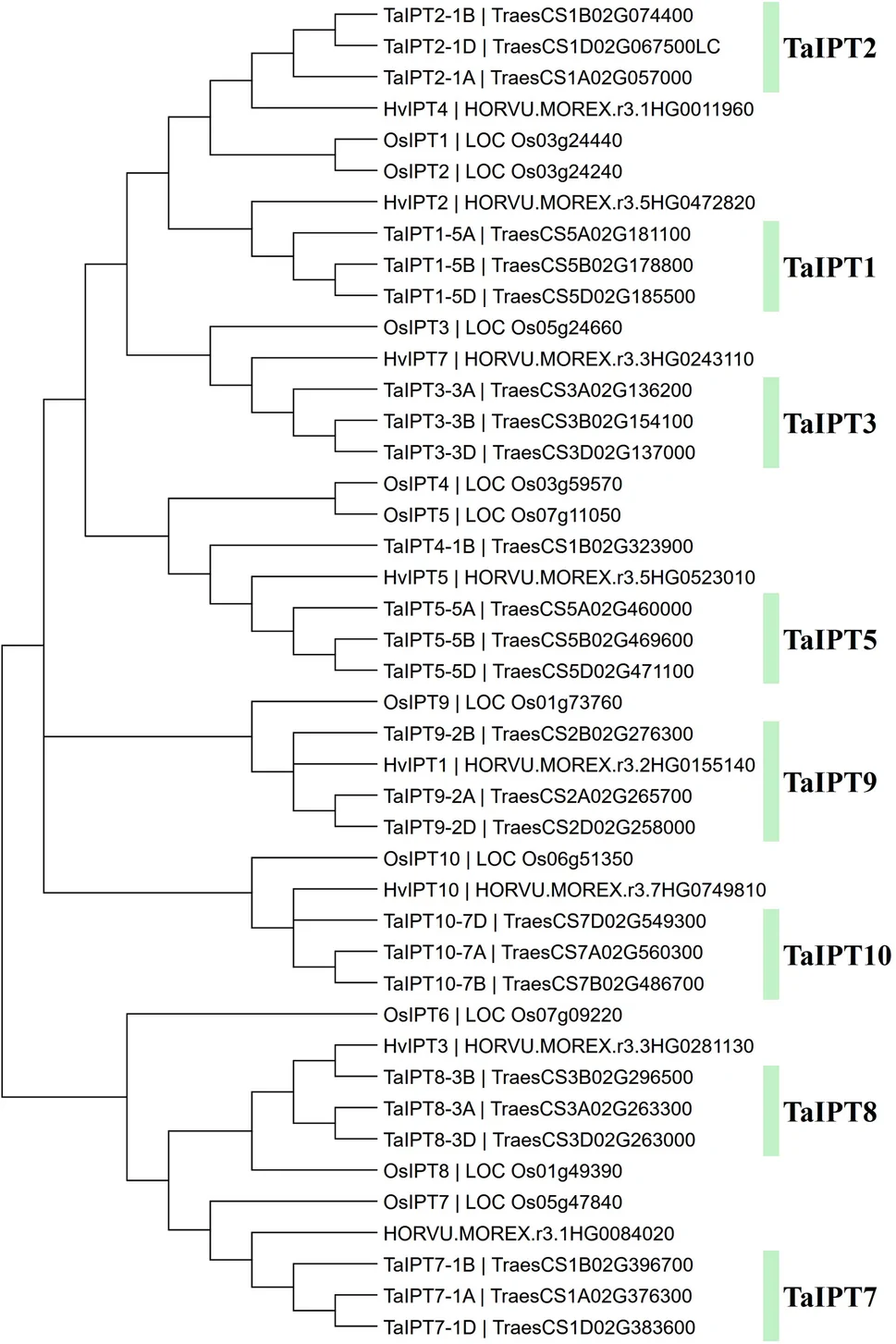
Phylogenetic framework of IPT proteins in cereals. Phylogenetic relationships among IPT proteins from *Triticum aestivum* (Ta), *Hordeum vulgare* (Hv), and *Oryza sativa* (Os). Protein sequences were retrieved from EnsemblPlants (*T. aestivum*,* H. vulgare*) and the Rice Genome Annotation Project (*O. sativa*). Evolutionary analysis was performed in MEGA12 [57] using the Neighbor-Joining method, and the consensus tree was inferred from 1000 bootstrap replicates. The phylogeny defines conserved and divergent IPT clades across cereals, providing an evolutionary framework for interpreting TaIPT structural diversity and functional specialization

*TaIPT* genes were identified via BLAST searches against the IWGSC RefSeq v1.1 and v2.1 assemblies using previously reported monocot *IPT* sequences as queries. Gene structures were examined *via* EnsemblPlants. Candidate sequences were retained when they contained the conserved IPT domain and showed reciprocal similarity to known cereal IPT proteins. Chromosomal positions, homoeologous relationships and predicted gene structures were checked in EnsemblPlants and compared with previous wheat IPT annotations [38, 44]. Rice (*Oryza sativa*) and barley (*Hordeum vulgare*) IPT sequences were included to support orthology-based nomenclature. Multiple sequence alignment was performed for full-length IPT proteins, and phylogenetic relationships were inferred in MEGA 12 using the Neighbor-Joining method with 1000 bootstrap replicates, as shown in Fig. 1.

### RNA extraction and cDNA synthesis

Total RNA was extracted with TRIzol Reagent (Invitrogen) according to the manufacturer’s protocol. RNA quality and integrity were assessed by agarose gel electrophoresis and spectrophotometric measurements, and RNA quantity was determined before reverse transcription. Potential genomic DNA contamination was minimized during RNA preparation and primer validation. First-strand cDNA synthesis was performed using the RevertAid First Strand cDNA Synthesis Kit (Thermo Scientific) with 2 µg RNA, following the validated workflow established for wheat developmental tissues [54]. The resulting cDNA was diluted to a uniform working concentration before qPCR.

### Reference gene selection and primer validation

Reference genes *Ref2* (*ADP-ribosylation factor*) and *Ta3006* (*Wings apart-like protein 2*) were selected based on their high stability across twelve wheat organs and both cultivars in Bocian et al. (2025). Primer specificity was confirmed by 2% agarose gels, single-peak melting-curve analysis, and PCR efficiency estimation using LinRegPCR 2021.2.

### RT-qPCR analysis

The qPCR primers for *TaIPT* genes were designed based on the sequences retrieved from the EnsemblPlants database (Table 1), and have been designed to target all the identified homoeologs of the gene simultaneously (Table S1); therefore, the RT-qPCR data represent gene-level expression rather than homoeolog-specific transcript abundance. This approach enabled robust comparison of total *TaIPT* gene activity across organs, developmental stages and normalization strategies, but it does not resolve A-, B- and D-subgenome-specific contributions. Primer specificity was confirmed by 2% agarose gels and melting-curve analysis.

RT-qPCR was performed on a CFX384 Touch Real-Time PCR Detection System (Bio-Rad Laboratories) using HOT FIREPol^®^ EvaGreen^®^ Mix. Reactions (10 µL) contained 4 µL diluted cDNA and 0.2 µM primers. Cycling conditions followed Bocian et al. [54]: 95 °C for 12 min; 50 cycles of 95 °C for 15 s, 60 °C for 15 s, 72 °C for 20 s; followed by a melt curve (70–99 °C). Three biological and three technical replicates were analyzed. Technical replicates were averaged before statistical analysis. Expression quantification used the standard-curve method. Normalization employed *Ref2*, *Ta3006*, or their geometric mean, consistent with validated normalization strategies for wheat developmental RT-qPCR analyses [54]. Mean-scaled relative expression values were used for visualization of spatiotemporal patterns, whereas absolute normalized expression values are provided in Table S2 to support cross-study comparison and assessment of transcript abundance differences among genes.

### Phytohormone extraction and quantification

Cytokinins: *trans*-zeatin (tZ), *trans*-zeatin riboside (tZR), *trans*-zeatin-9-glucoside (tZ9G), *trans*-zeatin-O-glucoside (tZOG), *cis*-zeatin (cZ), *cis*-zeatin-riboside (cZR), *cis*-zeatin O-glucoside (cZOG), *cis*-zeatin-9-glucoside (cZ9G) and abscisic acid (ABA) were extracted from 100 mg tissue using 50% acetonitrile. Extracts were purified by solid-phase extraction, evaporated, reconstituted, and quantified by UHPLC–MS/MS as described in Jablonski et al. [47]. Hormone concentrations were determined from three biological replicates per cultivar and developmental stage. The four analyzed spike stages (0, 4, 7 and 14 DAP) were used to capture major early reproductive transitions; finer temporal sampling will be required in future studies to identify transient hormone maxima or short-lived shifts in cytokinin-ABA balance.

### Statistical and correlation analyses

**Fig. 2:**
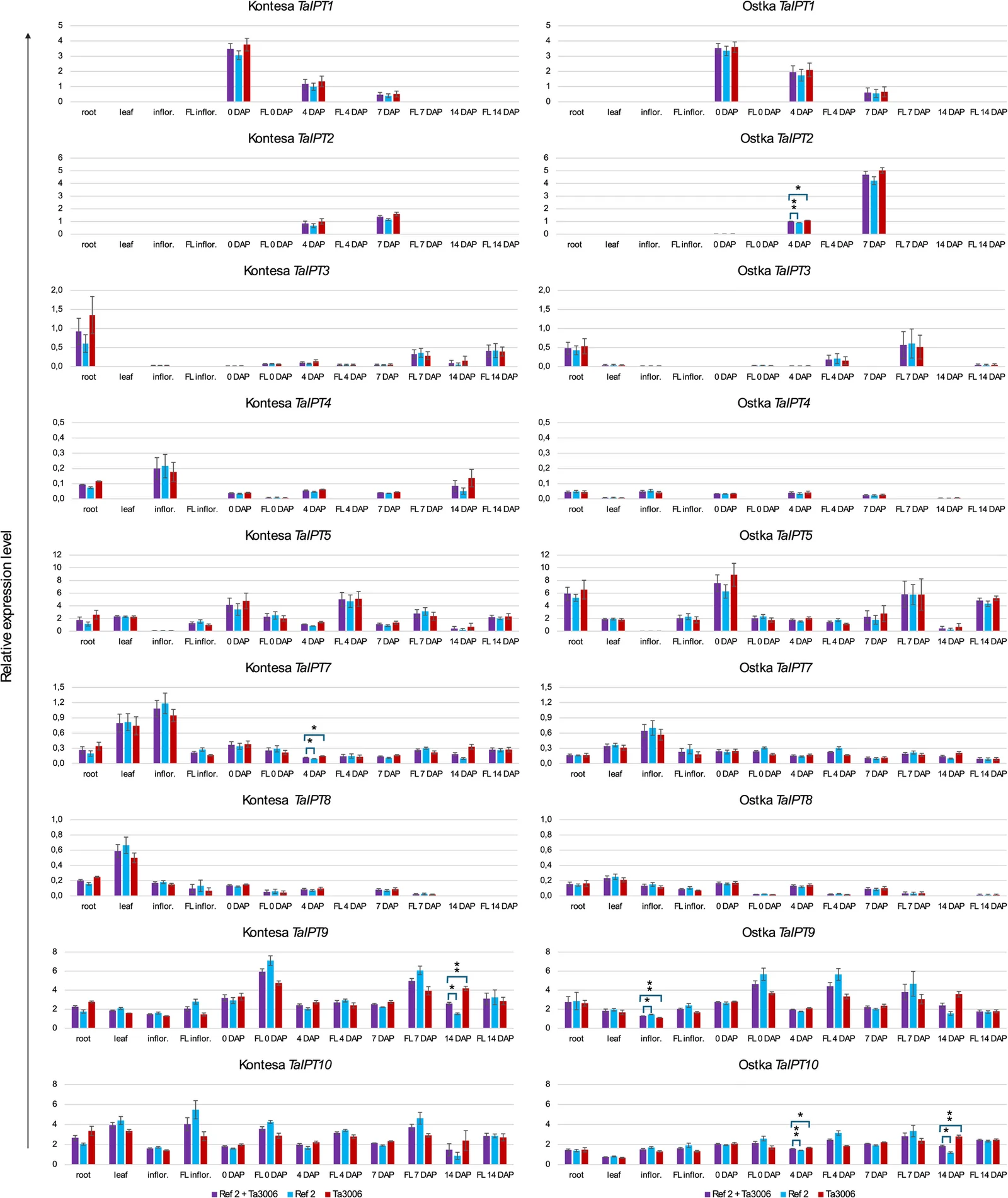
Spatiotemporal expression profiles of *TaIPT* genes. Expression profiles of *TaIPT* family genes across vegetative and reproductive tissues in wheat cultivars Kontesa (left panels) and Ostka (right panels). Values represent means from three biological replicates; technical qPCR replicates were averaged before analysis. Error bars indicate SE. The graphs show relative expression related to mean value of all samples in each group, normalized using *Ref 2*, *Ta3006*, or both reference genes; absolute normalized expression values are provided in Table S2. Asterisks indicate significant differences between the values obtained from single-gene and two-gene normalization strategies (*p < 0.05, **p < 0.01), based on the tests described in Materials and Methods. Distinct and cultivar-specific *TaIPT* expression modules highlight differential allocation of cytokinin biosynthetic activity between source and sink tissues during development

Statistical analyses were performed in Statistica 13.3. For RT-qPCR and hormone datasets, normality was tested with the Shapiro-Wilk test and homogeneity of variance with Levene’s test. For two-group comparisons, Student’s t-test was used when parametric assumptions were met, whereas the Mann-Whitney U test was used for non-parametric data. For comparisons involving more than two developmental stages or organs, one-way ANOVA followed by Bonferroni test was used for parametric data, and the Kruskal-Wallis test followed by appropriate post hoc comparisons was used for non-parametric data. For Figs. 2 and 4, values are presented as means from three biological replicates with variation indicated in the figure legends, and significance indicators are defined in the corresponding legends. Because expression and hormone datasets were not assumed to follow normal distributions and the expected relationships were monotonic rather than strictly linear, Spearman’s rank correlation coefficients (rho) were calculated for *TaIPT* transcript levels and hormone concentrations. Network figures show only associations that met both an effect-size threshold (|rho| >= 0.50, interpreted as at least a moderate association in this exploratory analysis) and a stringent p-value threshold (*p* < 0.01). These thresholds were used to reduce visual complexity and limit interpretation to stronger co-variation patterns. Correlations were interpreted as evidence of coordinated variation and not as proof of causal regulation.

## Results

### Identification and phylogenetic framework of the *TaIPT* gene family

Using current wheat genome assemblies and previous annotations [38], we identified nine core isopentenyltransferase genes (*TaIPT1*–*TaIPT10*) in bread wheat (*Triticum aestivum*). With the exception of *TaIPT4*, which is present only on chromosome 1B, all genes occurred as homoeologous triplets across the A, B, and D subgenomes, resulting in a total of 25 *TaIPT* homoeologs (Table 1). Gene nomenclature follows the standard convention combining gene identity and subgenome location (e.g. *TaIPT1-5 A*, *-5B*, *-5D*), enabling unambiguous comparison among homoeologs and across species.

**Table 1:**
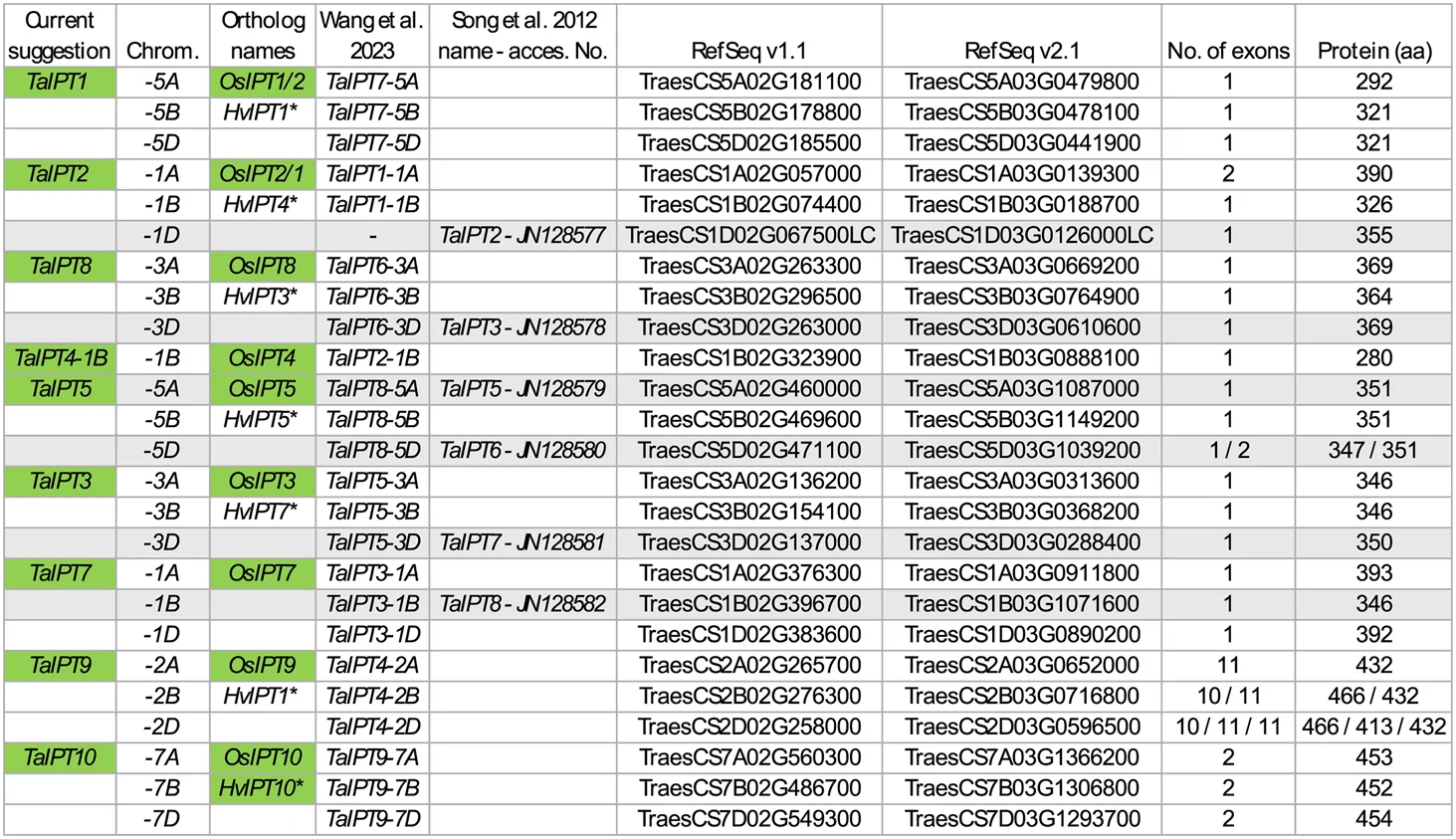
Identification and nomenclature of *TaIPT* family genes in bread wheat, their chromosomal locations, orthologs in rice and barley, and previous gene designations

* - according to Mrizova et al. 2013 [58]

Analysis of gene structure showed that most *TaIPT* genes consist of a single exon, consistent with other monocot *IPT* genes. In contrast, *TaIPT2-1 A*, *TaIPT5-5D*, and all three *TaIPT10* homoeologs contain two exons, while *TaIPT9* displays a markedly expanded structure comprising 11 exons, representing the most complex organization within the family.

Onthology-based comparisons confirmed conservation of *TaIPT* nomenclature and evolutionary relationships among wheat, rice (*Oryza sativa*) and barley (*Hordeum vulgare*) (Table 1). For example, *TaIPT1* corresponds to *OsIPT1/2*, *TaIPT2* to *OsIPT2/1*, and *TaIPT10* to *OsIPT10*/*HvIPT10*.

Phylogenetic analysis of IPT proteins from wheat, rice, and barley resolved TaIPTs into distinct evolutionary clades (Fig. 1). TaIPT9 and TaIPT10 formed branches separated from the remaining TaIPT members, reflecting sequence divergence within the gene family. Similar clustering patterns of *IPT9*- and *IPT10*-type genes have been reported in rice and barley.

### Spatiotemporal expression patterns of *TaIPT* genes

Transcript abundance of *TaIPT* genes was analyzed in vegetative and reproductive organs of two wheat cultivars, awnless Kontesa and awned Ostka (Fig. 2). Tissues included seedling roots, fully expanded leaves, immature inflorescences, spikes at anthesis (0 DAP), spikes at 4, 7, and 14 days after pollination (DAP), and the corresponding flag leaves. Slight variation was observed between absolute expression values among normalization strategies (*Ref2* + *Ta3006*, *Ref2*, *Ta3006*), however relative expression patterns were consistent across normalization approaches.

*TaIPT* genes displayed distinct spatial and temporal expression patterns across organs and developmental stages. *TaIPT1* and *TaIPT2* showed the strongest expression in reproductive tissues. *TaIPT1* transcripts were highest at anthesis (0 DAP) in both cultivars and decreased at later developmental stages. *TaIPT2* expression increased after anthesis, reaching the highest levels at 7 DAP, with three times higher transcript abundance in Ostka compared with Kontesa.

*TaIPT3*, *TaIPT4*, *TaIPT7*, and *TaIPT8* were expressed at relatively low to moderate levels across most organs. *TaIPT3* showed higher expression in seedling roots and moderate expression in flag leaves at later developmental stages. *TaIPT4* transcripts were detected mainly in roots and inflorescences of Kontesa. *TaIPT7* and *TaIPT8* exhibited relatively low expression in leaves and inflorescences.

*TaIPT5* transcripts were detected in multiple organs, including roots, spikes at anthesis, and flag leaves during early grain development. Expression levels of *TaIPT5* were generally higher in Ostka than in Kontesa.

*TaIPT9* and *TaIPT10* transcripts were detected in all analyzed tissues in both cultivars. *TaIPT9* showed relatively stable expression across organs, whereas *TaIPT10* exhibited somewhat higher transcript levels in vegetative tissues of Kontesa and similar to Ostka expression in reproductive tissues and subtending flag leaves.

### Organ-specific expression hierarchies of *TaIPT* genes

**Fig. 3:**
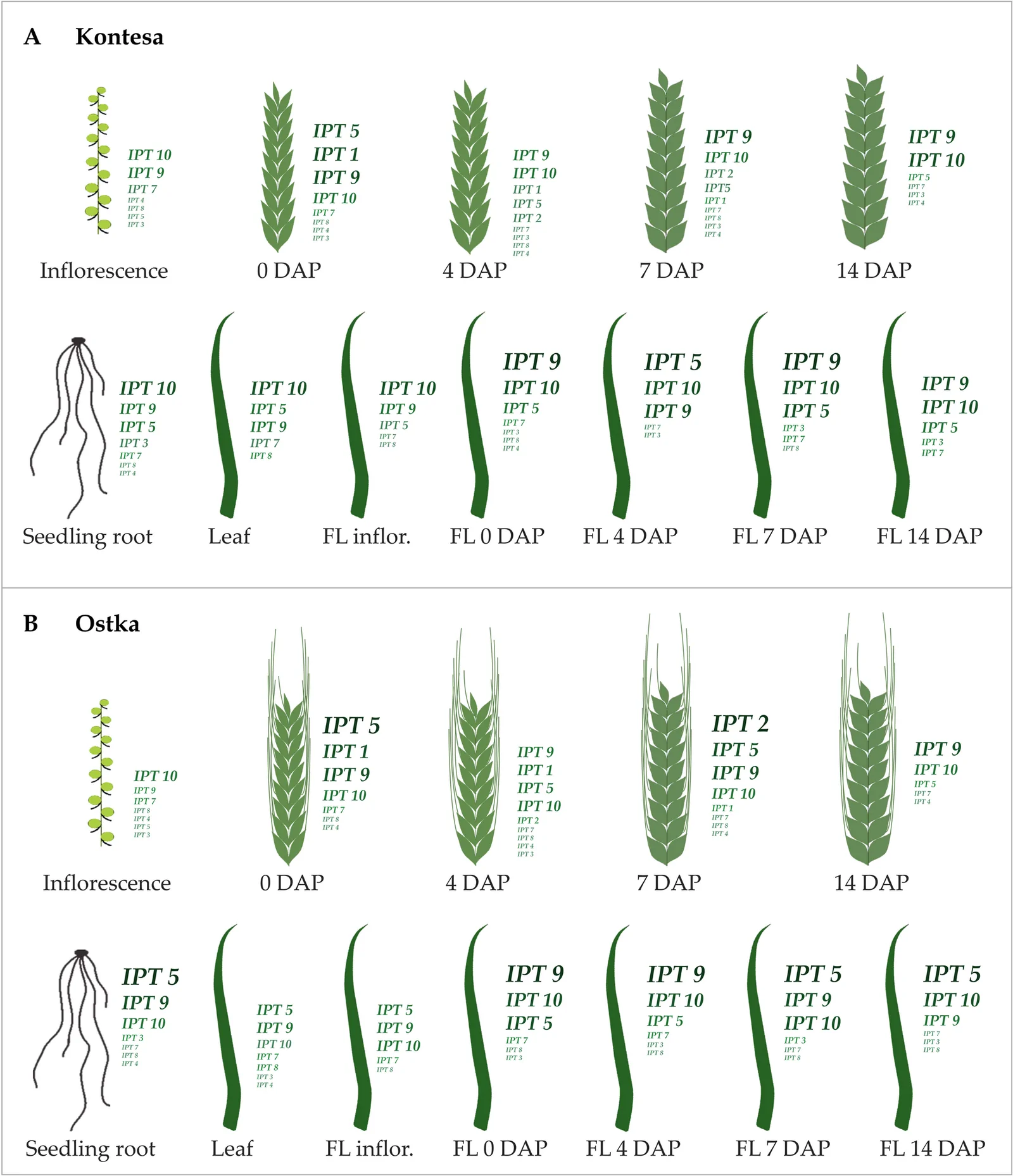
Organ-specific hierarchies of *TaIPT* expression. Graphical summary of *TaIPT* gene expression hierarchies in individual organs of wheat cultivars Kontesa (**A**) and Ostka (**B**). Organ-dependent ranking of transcript abundance reveals a modular organization of *TaIPT* genes, with broadly expressed and reproductive-specific isoforms contributing differentially to cytokinin homeostasis

Relative rankings of *TaIPT* transcript abundance across organs are summarized in Fig. 3. In seedling roots, *TaIPT9* and *TaIPT10* showed the highest transcript levels in both cultivars, while *TaIPT5* was more strongly expressed in Ostka.

In fully expanded leaves and pre-anthesis flag leaves, *TaIPT10* showed relatively high transcript abundance in Kontesa. In Ostka, higher expression levels were observed for *TaIPT5* and *TaIPT9*.

During anthesis and early grain development (0–14 DAP), several *TaIPT* genes were expressed in developing spikes. *TaIPT1* and *TaIPT5* showed relatively high transcript levels at anthesis, while *TaIPT9* and *TaIPT10* transcripts were detected throughout the analyzed stages. The differences between two cultivars are detected in 7 DAP spikes, where *TaIPT2* showed the highest transcript level in Ostka.

In pre-anthesis inflorescences, transcripts of *TaIPT7*, *TaIPT9*, and *TaIPT10* were detected at higher levels compared with other family members.

### Phytohormone dynamics during spike development

**Fig. 4:**
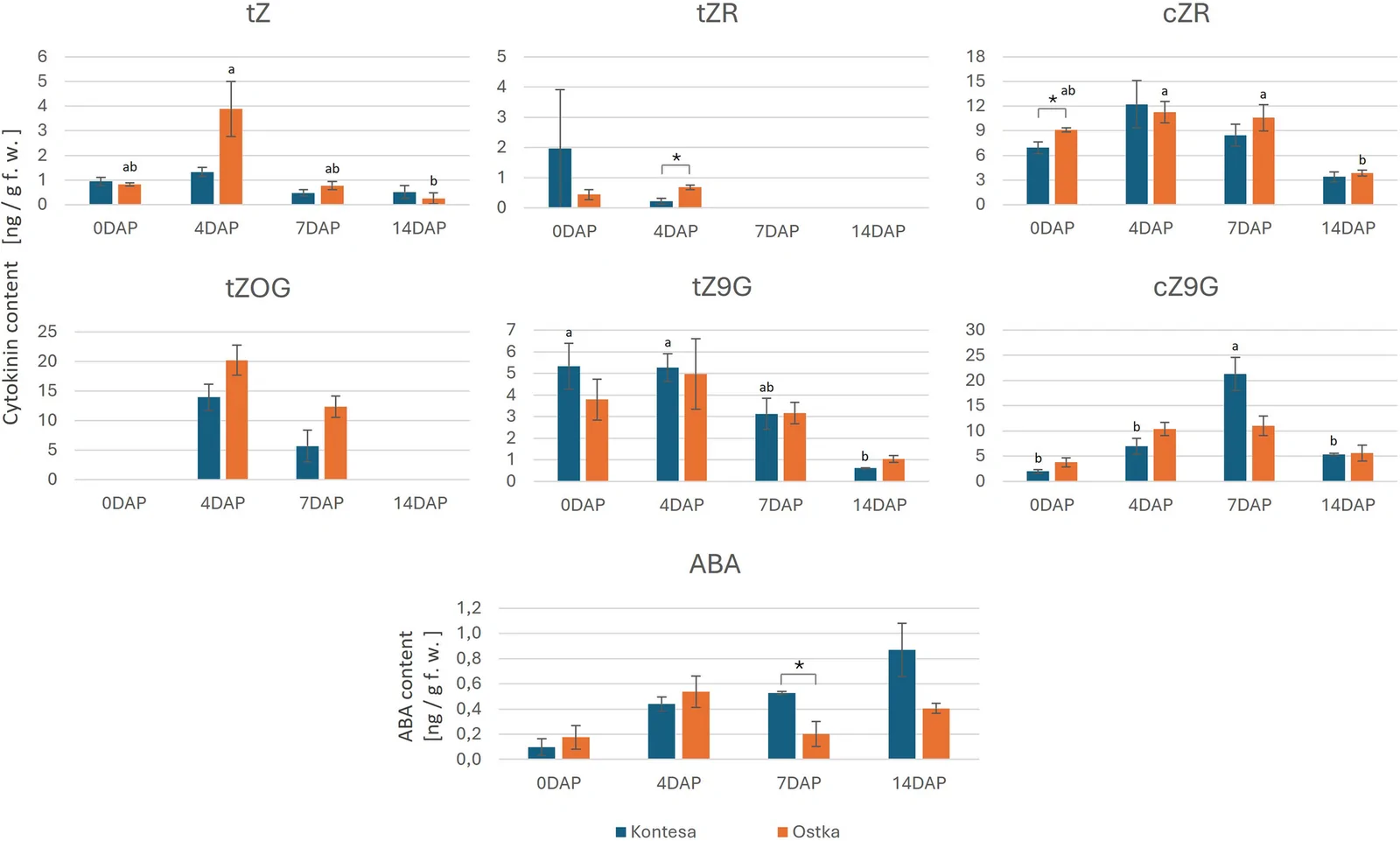
Developmental dynamics of phytohormones in wheat spikes. Concentrations of cytokinins and Abscisic Acid (ABA) in developing spikes of wheat cultivars Kontesa (blue bars) and Ostka (orange bars) at anthesis (0 DAP) and during early grain filling (4–14 DAP). Values are means +/- SD of three biological replicates. Different letters indicate significant differences among developmental stages within a cultivar, and asterisks indicate significant differences between cultivars at the same developmental stage (*p < 0.05, **p < 0.01), according to the statistical tests described in Materials and Methods. Hormone profiles demonstrate contrasting developmental strategies, with transient cytokinin accumulation in Kontesa and prolonged cytokinin dominance in Ostka during early reproductive development

Phytohormone concentrations were quantified in developing spikes of Kontesa and Ostka at anthesis (0 DAP) and during early grain development (4, 7, and 14 DAP) (Fig. 4). Measured compounds included active cytokinins, *trans*-zeatin (tZ), *trans*-zeatin riboside (tZR) and *cis*-zeatin riboside (cZR), cytokinin conjugates, tZOG, tZ9G and cZ9G, and abscisic acid (ABA).

Both cultivars showed developmental changes in cytokinin profiles during the analyzed period. The highest tZ concentration was observed at 4 DAP in Ostka, whereas lower values were detected at later stages. However, the difference between cultivars at this time point was not marked as statistically significant. In contrast, tZR showed a significant cultivar difference at 4 DAP, with higher levels in Ostka than in Kontesa. tZR was detectable mainly during the earliest stages and decreased strongly thereafter in both cultivars.

Among *cis*-zeatin derivatives, cZR showed high levels during early spike development. A significant cultivar difference was detected at 0 DAP, where Ostka accumulated more cZR than Kontesa. In both cultivars, cZR remained relatively high from 0 to 7 DAP and declined significantly by 14 DAP, indicating a developmental decrease during later early grain development.

Cytokinin conjugates also displayed stage-dependent variation. tZOG accumulated mainly at 4 and 7 DAP, with higher numerical values in Ostka than in Kontesa, although no significant cultivar differences were indicated. tZ9G was highest during the earliest stages and declined toward 14 DAP, particularly in Kontesa, where different letters indicate significant developmental differences. cZ9G showed a pronounced developmental peak in Kontesa at 7 DAP, significantly higher than earlier and later stages within this cultivar. In Ostka, cZ9G levels were more moderate and showed less pronounced developmental variation.

ABA increased during spike development, especially in Kontesa. A significant cultivar difference was detected at 7 DAP, where ABA concentration was higher in Kontesa than in Ostka. By 14 DAP, ABA remained numerically higher in Kontesa, supporting the tendency toward stronger ABA accumulation in this cultivar during later early grain development. Together, these hormone profiles indicate cultivar-dependent differences in cytokinin metabolism and ABA accumulation, with Ostka showing stronger early cZR and tZR accumulation, whereas Kontesa showed a more pronounced later increase in ABA and a marked cZ9G peak at 7 DAP.

### Correlation analysis between *TaIPT* expression and hormone concentrations

**Fig. 5:**
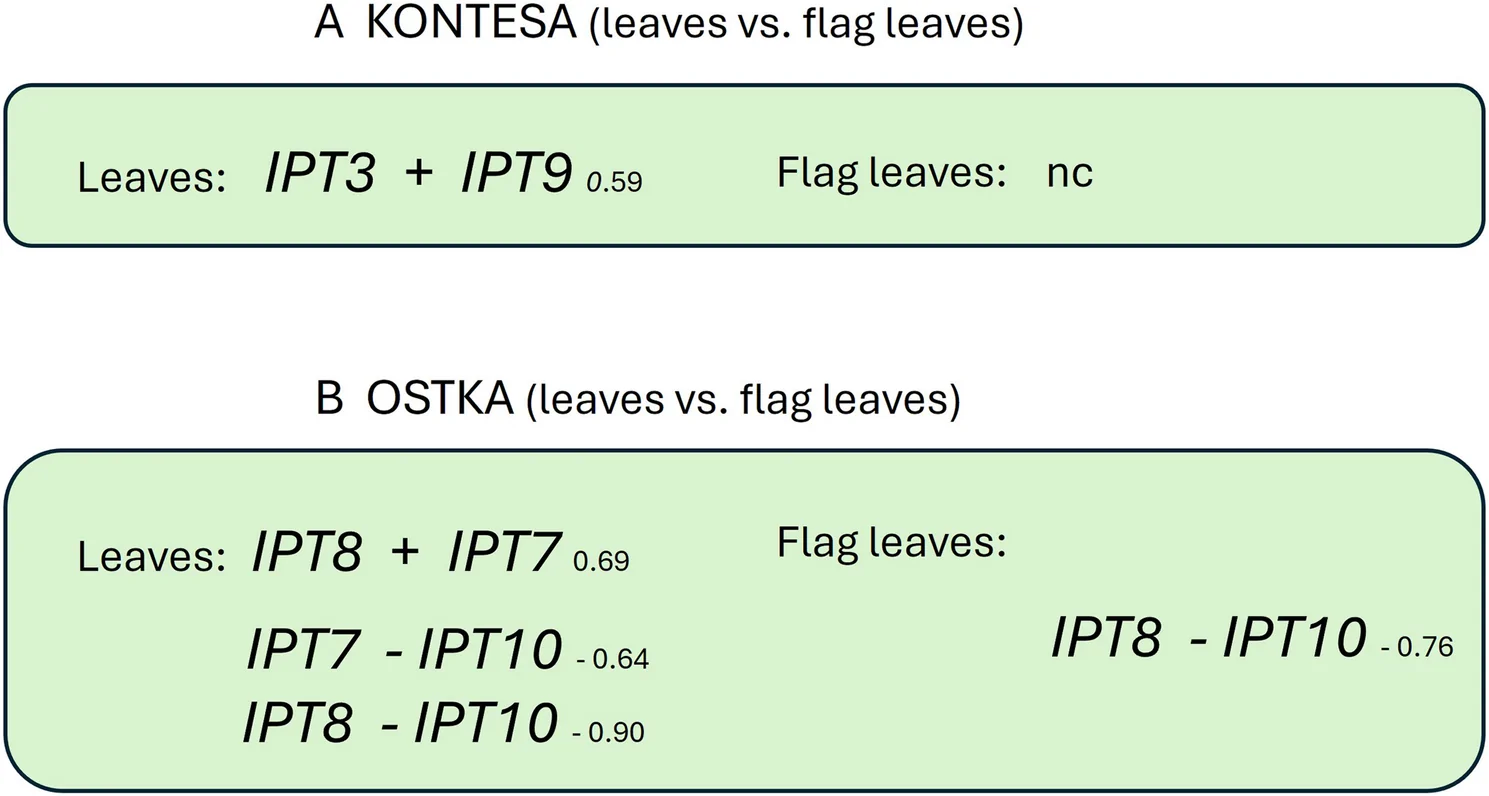
Spearman gene-to-gene correlation networks among *TaIPT* transcripts in vegetative tissues of wheat cultivars Kontesa (**A**) and Ostka (**B**). Correlations were calculated separately for all leaves, the group of flag leaves and seedling roots. Positive and negative associations are indicated by plus and minus signs, respectively, with Spearman’s rho values shown next to each retained association. Only correlations passing the filtering criteria described in Materials and Methods are shown. The absence of an association is indicated by nc (no correlation) or is not shown (seedling roots). The results indicate limited *TaIPT* coordination in Kontesa and stronger positive and negative relationships among selected *TaIPT* genes in Ostka leaves and flag leaves

**Fig. 6:**
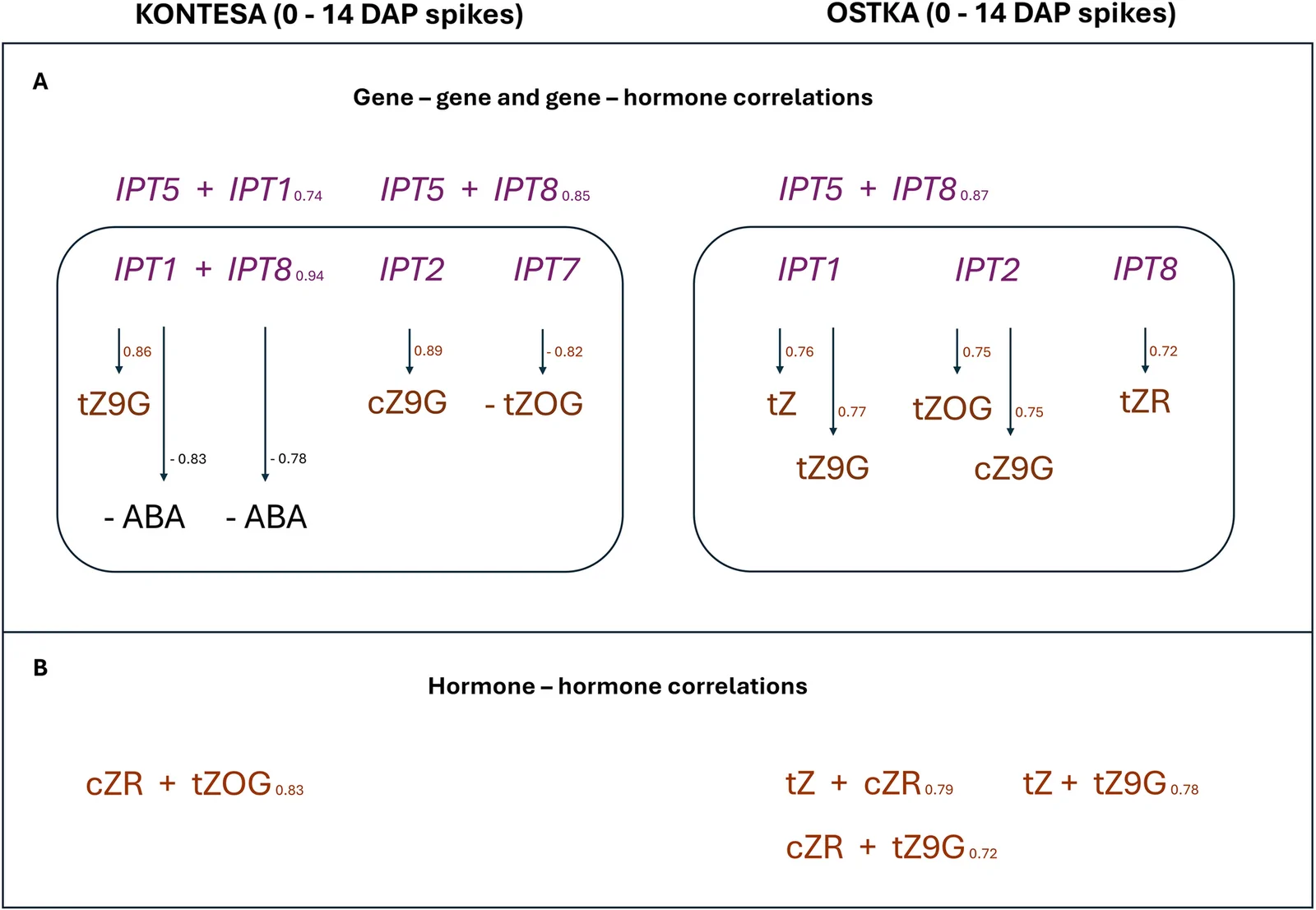
Spearman correlation networks among *TaIPT* transcripts and phytohormones in developing spikes of wheat cultivars Kontesa and Ostka from 0 to 14 DAP. **A** Gene-gene and gene-hormone correlations. **B** Hormone-hormone correlations. Positive and negative associations are indicated by plus and minus signs or arrows, and Spearman’s rho values are shown next to each retained association. Only correlations passing the filtering criteria described in Materials and Methods are shown. The figure combines the information previously presented separately as gene-hormone and hormone-hormone networks and should be interpreted as evidence of coordinated variation, not causality

To investigate potential coordination among *TaIPT* biosynthetic genes and their relationship with cytokinin and ABA metabolism, Spearman’s rank correlation analyses were performed using transcript abundance and hormone concentration data obtained from vegetative tissues (seedling roots, leaves, and flag leaves subtending spikes at 0–14 DAP) and reproductive tissues (immature inflorescences and spikes at 0–14 DAP) of wheat cultivars Kontesa and Ostka (Figs. 5 and 6). Spearman’s rho and nominal p-values were calculated. To reduce over-interpretation of weak or unsupported associations, only correlations meeting both |rho| >= 0.50 and *p* < 0.01 were retained in the network figures. These networks were interpreted as patterns of coordinated variation, not as evidence of direct causal regulation.

### Gene–gene correlation in vegetative tissues

Spearman correlation analysis of *TaIPT* expression in vegetative organs revealed cultivar- and organ-specific differences in transcriptional coordination (Fig. 5).

In Kontesa, only one positive association was retained in the pooled leaf dataset, between *TaIPT3* and *TaIPT9* (rho = 0.59). No significant *TaIPT* gene-gene correlations were detected in the group of flag leaves or seedling roots after filtering, indicating weak or highly compartmentalized co-variation of *TaIPT* transcription in the analyzed vegetative tissues.

In Ostka, the vegetative *TaIPT* network was more structured. In the pooled leaf dataset, *TaIPT8* was positively correlated with *TaIPT7* (rho = 0.69), whereas *TaIPT10* showed negative correlations with *TaIPT7* (rho = -0.64) and *TaIPT8* (rho = -0.90). In the group of flag leaves, a negative association between *TaIPT8* and *TaIPT10* was also retained (rho = -0.76), while seedling roots showed no retained correlations. These patterns indicate stronger positive and negative co-variation among selected *TaIPT* genes in photosynthetically active organs of Ostka.

### Gene-gene, gene-hormone and hormone-hormone correlations in developing spikes

Correlation analysis in 0–14 DAP spikes revealed distinct cultivar-specific networks linking *TaIPT* transcripts with cytokinin and ABA dynamics (Fig. 6A). In Kontesa, a positive gene-gene module was detected among *TaIPT5*, *TaIPT1* and *TaIPT8*, including positive associations between *TaIPT5* and *TaIPT1* (rho = 0.74), *TaIPT5* and *TaIPT8* (rho = 0.85), and *TaIPT1* and *TaIPT8* (rho = 0.94). *TaIPT1* was positively associated with tZ9G (rho = 0.86), and negatively associated with ABA (rho = -0.83). Additional Kontesa-specific associations included a negative correlation between *TaIPT8* and ABA (rho = -0.78), a positive correlation between *TaIPT2* and cZ9G (rho = 0.89), and a negative correlation between *TaIPT7* and tZOG (rho = -0.82).

In Ostka spikes, the retained gene-gene network included a positive association between *TaIPT5* and *TaIPT8* (rho = 0.87). Gene-hormone correlations linked *TaIPT1* with tZ (rho = 0.76), and tZ9G (rho = 0.77), *TaIPT2* with tZOG (rho = 0.75) and cZ9G (rho = 0.75), and *TaIPT8* with tZR (rho = 0.72). These associations indicate coordinated variation among selected *TaIPT* transcripts and active, riboside or conjugated cytokinin forms during early spike development, without implying direct regulatory causality.

Hormone-hormone correlations in the same 0–14 DAP spike dataset further differentiated the cultivars (Fig. 6B). Kontesa retained a single positive association between cZR and tZOG (rho = 0.83), whereas Ostka showed a small but more interconnected cytokinin module, including positive associations between tZ and cZR (rho = 0.79), tZ and tZ9G (rho = 0.78), and cZR and tZ9G (rho = 0.72). Together, the Spearman-based networks support a more restricted cytokinin-*TaIPT*-ABA interaction pattern in Kontesa and a more coordinated cytokinin network in Ostka.

## Discussion

### Evolutionary organization of *TaIPT* genes in wheat

The present study integrates genomic identification, developmental expression profiling and hormone quantification to refine the functional interpretation of the wheat *TaIPT* gene family. Nine core *TaIPT* genes represented by 25 homoeologs were confirmed across the A, B, and D subgenomes. While the existence of wheat *IPT* genes has been reported previously [38, 44], the added value of this study is the combination of a unified *TaIPT* nomenclature with organ- and stage-resolved expression and cytokinin/ABA profiling in two contrasting cultivars. This approach shows that the same gene family can be embedded in different transcriptional-hormonal networks depending on cultivar background.

Genome-wide analyses of cytokinin-related gene families in wheat and other cereals have revealed diversification and subfunctionalization of CKX, LOG, NAC and cytokinin conjugation enzymes, indicating that cytokinin homeostasis relies on coordinated regulation of biosynthesis, activation, degradation, conjugation and transcriptional control pathways [22, 23, 28, 53, 59–61]. In this context, the *TaIPT* family represents the biosynthetic entry point. Its cultivar-dependent expression provides insight into how cytokinin production may be associated with downstream metabolism and hormonal crosstalk during reproductive development.

Phylogenetic relationships separated TaIPTs into the two major IPT clades described across angiosperms: ATP/ADP-dependent IPTs and tRNA-dependent IPTs [17, 26, 35, 55]. This distinction is biologically important because ATP/ADP-IPTs are expected to contribute more directly to pools of active isoprenoid cytokinins, whereas tRNA-IPTs mainly support *cis*-zeatin production through tRNA turnover [22, 62]. Therefore, stable expression of *TaIPT9* and *TaIPT10* most likely reflects constitutive metabolic activity rather than direct control of short developmental cytokinin peaks.

### Developmental regulation of *TaIPT* genes during spike growth

Expression profiling revealed clear spatial and temporal differentiation among *TaIPT* genes. *TaIPT1* and *TaIPT2* were preferentially expressed in reproductive tissues, especially around anthesis and early grain development, consistent with the role of cytokinins in meristem activity, spikelet development and early endosperm cell proliferation [4, 33, 44, 63].

In contrast *TaIPT3*, *TaIPT4*, *TaIPT7* and *TaIPT8* showed lower or broader expression, suggesting functions in basal cytokinin supply. *TaIPT5* was detected in both vegetative and reproductive organs, supporting a possible link between source-tissue cytokinin production and developing sinks [5]. Because the expression profiles are based on gene-level primers that amplify homoeologous copies together, future homoeolog-specific assays or RNA-seq analyses will be needed to resolve subgenome-specific contributions.

### Cytokinin dynamics, ABA balance and cultivar-specific strategies

Hormone profiling confirmed that early spike development is accompanied by dynamic changes in cytokinin pools. Both cultivars showed transient accumulation of active cytokinins and cytokinin conjugates around anthesis and early grain filling, in agreement with studies linking spike cytokinin status with grain-number traits and yield potential in wheat [6, 7]. The subsequent increase in ABA is consistent with the transition from cell division and sink establishment toward maturation, dehydration and senescence-associated processes [12, 64].

However, the four sampled time points provide only a broad developmental profile. They are sufficient to reveal major differences between cultivars but cannot define the precise timing of short-lived cytokinin peaks or rapid cytokinin-ABA transitions.

The comparison between awnless Kontesa and awned Ostka provides a useful but intentionally limited framework for interpreting cultivar-specific hormone patterns. Kontesa represents an older awnless bread-quality spring wheat cultivar with early heading and maturity and moderate yield potential relative to newer cultivars, whereas Ostka Smolicka is an awned quality cultivar with high grain-quality parameters, good lodging and sprouting resistance, and tolerance to spring frost. These agronomic differences help define the biological context of the comparison, but they also mean that the observed molecular and hormonal differences cannot be attributed to awns alone. Ostka showed significantly higher cZR at anthesis and higher tZR at 4 DAP, together with a numerical increase in tZ at 4 DAP and stronger *TaIPT2* expression at 7 DAP, suggesting enhanced early cytokinin-related activity. In contrast, Kontesa showed a pronounced cZ9G peak at 7 DAP and significantly higher ABA at 7 DAP, supporting an earlier or stronger transition toward ABA-associated maturation. Because awns are photosynthetically active organs and can contribute assimilates to developing grains, the awned phenotype of Ostka may be associated with a more prolonged cytokinin-supported source-sink phase. This interpretation is consistent with reports that awn-related loci affect grain length in wheat [65] and that cytokinin activation can influence both awn length and grain production in rice [32].

Importantly, the present experiment was not designed to test awn function directly, and Kontesa and Ostka are not near-isogenic lines. Therefore, the observed differences cannot be attributed solely to awn morphology; they may also reflect broader differences in cultivar background, phenology, plant architecture or source-sink capacity. Because photosynthetic rate, assimilate transport and grain sink capacity were not measured, the combined expression and hormone data support a working model rather than a causal conclusion: Kontesa appears to undergo a more rapid transition from cytokinin-dominated early development to ABA-associated maturation, whereas Ostka maintains a more coordinated cytokinin co-variation network during the early post-anthesis period. Future experiments combining awn removal, high-resolution developmental sampling, photosynthetic measurements and grain-number or grain-weight data would be valuable to test this model directly.

### Correlation networks linking *TaIPT* expression with cytokinin and ABA metabolism

The Spearman-based correlation networks refined the interpretation of cultivar-dependent *TaIPT* coordination while also emphasizing the limits of the dataset. In vegetative tissues, Kontesa retained only one positive association, *TaIPT3*-*TaIPT9* in the pooled leaf dataset, and no retained correlations in flag leaves or seedling roots. In contrast, Ostka showed a more organized leaf and flag-leaf network, characterized by a positive *TaIPT7*-*TaIPT8* relationship and negative associations involving *TaIPT10*. These patterns suggest that *TaIPT* transcription in Kontesa is more weakly coordinated or organ-specific, whereas Ostka maintains stronger opposing co-variation between selected *TaIPT* genes in photosynthetically active tissues.

In developing spikes, the two cultivars also differed in the structure of *TaIPT*-hormone relationships. Kontesa showed a *TaIPT5*-*TaIPT1*-*TaIPT8* module associated with tZ9G and negative co-variation with ABA, as well as additional *TaIPT2*-cZ9G and *TaIPT7*-tZOG relationships. This pattern is consistent with a cytokinin module that is partly separated from, and in some cases negatively associated with, ABA accumulation. In Ostka, *TaIPT5* and *TaIPT8* were positively associated, and *TaIPT1*, *TaIPT2* and *TaIPT8* were linked with tZ and tZOG, tZOG and cZ9G and tZR, respectively. The hormone-hormone network in Ostka connected tZ, cZR and tZ9G, indicating coordinated variation among cytokinin forms during early spike development. These results support the conclusion that Ostka maintains a more integrated cytokinin co-variation network, whereas Kontesa shows a more restricted network with clearer negative associations involving ABA. Because these analyses are correlative, they should be interpreted as hypotheses for future functional validation rather than as evidence that specific *TaIPT* genes directly control hormone concentrations.

A logical functional follow-up would be to prioritize two complementary groups of *TaIPT* genes. The first group includes reproductive- and spike-associated candidates such as *TaIPT1*, *TaIPT2* and *TaIPT8*, which showed reproductive expression and/or contributed to spike correlation modules in the present study. The second group includes broadly expressed candidates such as *TaIPT9* and *TaIPT10*, which showed high transcript abundance across multiple organs, particularly in seedling tissues and corresponding flag leaves, suggesting possible roles in basal cytokinin metabolism or source-tissue cytokinin homeostasis. *TaIPT5* should be considered separately because, in our nomenclature, it corresponds to *TaIPT8* in Wang et al. [38], where it was already functionally characterized in relation to cytokinin levels and drought tolerance; thus, it represents a validated comparative reference and a candidate for reproductive-stage analysis rather than a completely novel functional target. Future work should generate homoeolog-specific or combined A/B/D genome-edited lines for selected reproductive and broadly expressed *TaIPT* candidates, select homozygous edited genotypes, and quantify cytokinin metabolites, ABA, spike development, awn traits, flag-leaf function, and grain-number or grain-weight components across high-density developmental time courses. Complementary transient expression, overexpression or RNA interference approaches, together with homoeolog-specific RT-qPCR or RNA-seq, would help separate total gene-level effects from subgenome-specific contributions.

### Integration with nitrogen-responsive regulatory networks

Cytokinin homeostasis is not determined by *TaIPT*-mediated biosynthesis alone. Active cytokinin levels also depend on LOG-mediated activation, CYP735A-dependent hydroxylation, CKX-mediated degradation, cytokinin conjugation and transcriptional regulation by factors such as NAC proteins [28, 47–53, 59, 60]. Therefore, the *TaIPT* expression patterns reported here cannot fully explain the endogenous cytokinin profiles by themselves, but should be interpreted as one component of a broader cytokinin metabolic network.

Nitrogen availability provides an additional context because cytokinins act as systemic signals coordinating nutrient status with shoot growth and reproductive development [5, 55, 66]. Although related datasets from these cultivars suggest genotype-specific regulation of hormone-associated pathways, those data are not part of the present study and are therefore not used as direct evidence here. Recent advances in single-cell and spatial transcriptomics further show that plant responses to environmental cues are strongly cell-type-specific [56]. This is relevant for cytokinin biology because bulk organ-level measurements, such as those used here, average signals from multiple tissues and cell types. Cell-type-resolved approaches could therefore help identify where *TaIPT* transcripts and cytokinin changes originate within developing spikes and flag leaves.

Together, these results place *TaIPT*-mediated cytokinin biosynthesis within a broader regulatory network involving cytokinin activation, degradation, conjugation and transcriptional control. The main biological insight of the present study is that cultivar background modifies the co-variation between *TaIPT* expression and hormone profiles during reproductive development. Thus, the *TaIPT* family should not be interpreted only as a static set of biosynthetic genes, but as part of a genotype-dependent hormonal system whose physiological relevance requires future cross-pathway and functional validation.

## Conclusions

This study provides an integrated genomic, transcriptional, and hormonal analysis of the *TaIPT* gene family during wheat reproductive development. By combining a unified *TaIPT* nomenclature with developmental expression profiling and cytokinin/ABA quantification, the work defines cultivar-dependent transcriptional-hormonal association patterns in awnless Kontesa and awned Ostka.

The main insight is that *TaIPT* expression is not organized as a uniform module across cultivars. Kontesa showed more restricted *TaIPT*-hormone co-variation and stronger ABA accumulation at 7 DAP, whereas Ostka showed a more coordinated cytokinin-related network during early spike development. These differences may be relevant to source-sink coordination and cultivar adaptation, but this interpretation remains hypothetical and should be considered as cultivar-background dependent rather than an isolated effect of awn morphology, because photosynthesis, assimilate transport, sink capacity and functional gene effects were not directly tested.

The study is descriptive and correlative, and direct functional validation will be required to establish causality. Nevertheless, the dataset identifies candidate *TaIPT* genes, developmental stages and cultivar-specific hormonal relationships that provide a foundation for future genome editing, homoeolog-specific expression analyses, cross-pathway cytokinin studies and cell-type-resolved analyses of wheat reproductive development.

## Supplementary Information

Supplementary Material 1: Table S1. Reference genes and primers used in this study. Table S2. Raw qPCR results and absolute value normalization in TaIPT genes expression analysis.

## Data Availability

All data generated or analyzed during this study are included in this published article and its supplementary information files.
